# Understanding Immune Responses to Surgical Transplant Procedures in Stevens Johnsons Syndrome Patients

**DOI:** 10.3389/fmed.2021.656998

**Published:** 2021-05-21

**Authors:** Matias Soifer, Hazem M. Mousa, Robert B. Levy, Victor L. Perez

**Affiliations:** ^1^Foster Center for Ocular Immunology, Duke Eye Institute, Durham, NC, United States; ^2^Department of Ophthalmology, Duke University Medical Center, Durham, NC, United States; ^3^Department of Microbiology and Immunology, University of Miami Miller School of Medicine, Miami, FL, United States

**Keywords:** Stevens Johnsons, corneal transplant, immunosuppression, limbal stem cell transplant, high risk corneal transplantation

## Abstract

Stevens Johnsons syndrome (SJS) is a mucocutaneous disorder caused by an autoimmune response most commonly to medications. Unless it is properly managed in the acute setting, this entity can affect the ocular surface causing chronic cicatrizing conjunctivitis with limbal stem cell deficiency and lid anomalies which ultimately result in corneal opacities that may limit patients' visual acuity. When this stage is reached, some patients might need to undergo some form of corneal and/or limbal stem cell transplantation that exposes an already sensitized immune system to a new alloantigen. While the innate immunity plays a role in corneal graft survival, adaptive immune responses play a major part in corneal graft rejection and failure, namely through CD4+ T cell lymphocytes. Hence, the management of the immune response to surgical transplant procedures in SJS patients, involves a dual approach that modulates the inflammatory response to a new alloantigen in the context of an autoimmune sensitized patient. This review will explore and discuss current perspectives and future directions in the field of ocular immunology on how to manage SJS immune responses to ocular surgical procedures, reviewing systemic and local immunosuppressive therapies and protocols to adequately manage this debilitating condition.

## Introduction

Stevens Johnsons syndrome (SJS) is a vesiculobullous disorder that affects the mucocutaneous tissues, which are generally triggered by an autoimmune response to different medications, commonly cold medications, anticonvulsants, and sulfa drugs (1). This condition has classically been defined as a spectrum of disease, called SJS when there is <10% of skin involvement and toxic epidermal necrolysis (TEN) when it is more than 30%. For the purposes of this review, SJS will account for the entire spectrum of the disease. The overall incidence for this disorder has been estimated as 0.5-1.89 per million inhabitants (2–4). SJS is diagnosed clinically as a skin reaction with epidermal necrolysis in conjunction with the histopathological findings of subepidermal blisters and apoptotic keratinocytes. This is presumed to occur as a result of T-cell mediated type IV hypersensitivity reaction (5), on which CD14+ monocytes and CD4 T cells contribute to the activation of the effector CD-8+ T cells (5–7), which mediate cell death through induction of apoptosis. This entity affects the ocular surface with a frequency of 40-75% of cases acutely (8), and about 63% of SJS patients present late symptoms of ocular surface involvement (9). During the acute episode, the classical finding is a bilateral conjunctivitis that ranges from simple hyperemia to widespread sloughing of the ocular surface, tarsal conjunctiva, and lid margins. The acute complications typically resolve within 2-4 weeks; however, conjunctival scarring may result from the initial inflammatory course. In order to prevent this, it is important to perform an early amniotic membrane transplantation (AMT) to the ocular surface, since this procedure has been shown to be associated with better outcomes (10). Importantly, a longitudinal 10 year review of 284 pediatric patients with SJS, revealed that 99% did not receive AMT during the acute setting, and 60% of these developed low vision or blindness (11). Common chronic sequelae from ocular SJS are entropion, trichiasis, and instability of the tear film that set up a vicious cycle of slow persistent inflammation, which provokes constant injuries to the ocular surface leading to corneal scarring, keratinization, and blink related trauma, further damaging the cornea, conjunctiva, and limbal stem cells, which ultimately may limit patients' visual acuity. Because of the corneal opacification resulting from the described mechanisms, some patients might need to undergo corneal transplant to increase visual acuity, in combination with another allogeneic stem cell transplant, keratolimbal allograft (KLAL). These procedures represent a challenge in SJS patients, since the corneal transplants are considered high risk due to their corneal neovascularization, dry ocular surface, and lid anomalies. Moreover, since the involvement is often bilateral, there is no possibility of performing an autologous “non-allogeneic” transplant from the contralateral eye. Therefore, the ocular surface reconstruction of SJS patients with allogeneic tissue, represents an immunological challenge as they have already had an autoimmune response to ocular surface “auto-antigens.” Hence, the management of the immune response to transplant procedures in SJS patients involves a dual approach that modulates the inflammatory response to a new alloantigen in the context of an autoimmune sensitized patient. These procedures require a thoughtful and targeted systemic immunosuppression to avoid graft rejection, which includes modulation of the alloimmune and autoimmune responses. In order to understand the current perspectives on the immunologic approach to limbal and corneal transplants on SJS we conducted a literature review of publications from prestigious journals based on updated studies of ocular Stevens Johnsons and the immunologic management of corneal transplants and ocular surface reconstruction.

## Sjs Patients Are High Risk Corneal Transplant Recipients

In a low-risk PK, defined as a naive transplant on a cornea without neovascularization on a non-inflamed host bed, only a minority of transplants will experience an immune reaction and these may rarely lead to graft failure. These successful outcomes are based on a quick inhibition of the vascular sprouting following surgery, that restores the normal angiogenic privilege of the cornea (12), which explains the excellent prognosis of low-risk PK with a survival of 90% of grafts at 1 year (13). Importantly, management of these corneas does not require systemic immunosuppression and tapered topical steroid therapy are generally enough to avoid graft rejection. This is not the case for SJS patients. These patients are considered to be high-risk PK recipients, not only because of corneal neovascularization but also because the lymphatic system invades the cornea. These offer a continuous delivery of immune effector cells to the graft and favors migration of antigen presenting cells to lymphoid tissues, particularly in the neck, activating T cell lymphocytes and ultimately causing transplant rejection (14, 15). Hence, on SJS patients undergoing corneal transplants, systemic immunosuppression is required to dampen the aggressive inflammatory response. Fundamentally, the anti-inflammatory therapy must be directed toward the adaptive immunity, essentially T cell lymphocytes, which have been shown to participate in allografts rejection in other organs (16) and the cornea (17). Therefore, similarly to solid organ transplantation, SJS high risk corneal transplants associated with limbal stem cell grafting requires the use of three groups of medications to prevent graft rejection: these are mainly the use of a systemic T-cell inhibitor in combination with an antimetabolite, and an early short course of steroid.

## Targeting Ocular Alloimmune Responses In Sjs

**Figure 1:**
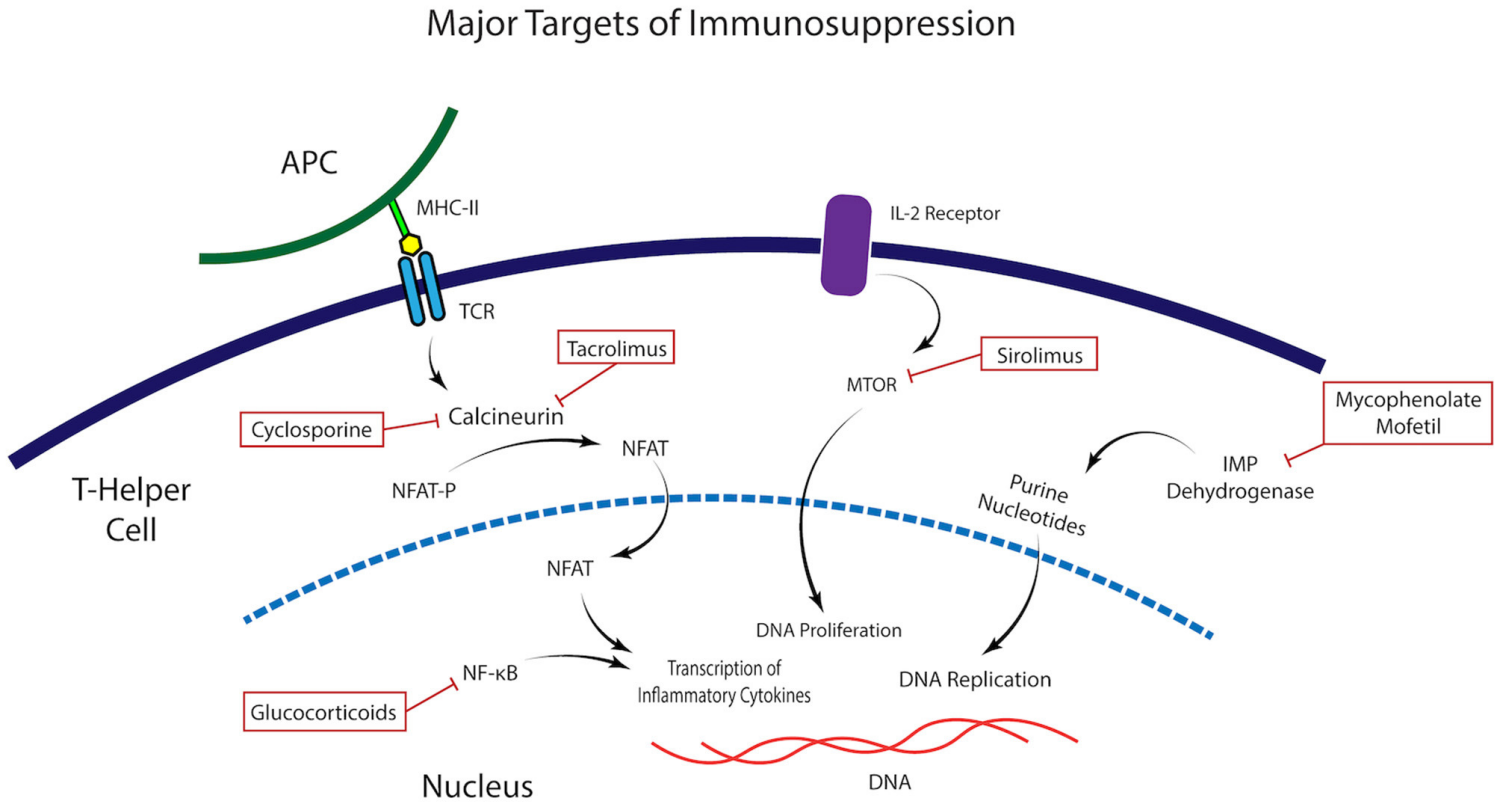
Molecular targets of immunomodulatory medications. Illustration of the mechanism of action and molecular targets of the immunomodulatory agents commonly used in the control of the alloimmune and autoimmune response in Stevens Johnsons Syndrome.

To inhibit the immunologic response directed to and elicited by the antigens carried in the donor graft, the pivotal elements of a chronic immunomodulation are the calcineurin inhibitors, which inhibit T cells activation. These are Cyclosporine, Tacrolimus, and Sirolimus and should be started at the time of transplantation (Figure 1). The rationale for their use is to provoke an immunosuppression that stops one or many steps in the path that is started when the donor antigens from the transplant are presented to the T-cell receptor and trigger the immune response that involves interleukin-2 (IL-2) and other factors, eventually leading to T-cell proliferation, migration, and attack on the corneal or limbal graft. Cyclosporine has been studied on patients with total limbal stem cell deficiency (LSCD) undergoing KLAL with a mean of 34 months of follow up. Fifty three eyes, of which nine had a history of SJS, received 5 mg/kg of oral cyclosporine daily. On follow up, the overall ambulatory vision (vision >20/200) decreased with time, being 44.6% at 5 years. Notably, SJS was the group with worse survival of ambulatory vision and survival of PK (18). This suggests that for SJS patients, cyclosporine is not an optimal choice for immunosuppression. In contrast, Tacrolimus success has been reported in KLAL in six patients with LSCD with 11 months of mean follow up (19). A group from the Cincinnati Eye Institute Cincinnati presented an immunosuppression protocol to address limbal transplantation (20), on which they administered high-dose oral corticosteroids, in addition to oral tacrolimus initiated at 4 mg twice daily and oral mycophenolate mofetil 1 g twice daily. With this protocol, after a mean follow-up of 62.7 months, the authors achieved an incidence of rejection of 31.1% which was lower to other studies using only oral cyclosporine (18, 20). This same group evaluated their protocol in 19 patients with severe ocular surface disease undergoing living related conjunctival limbal allograft (CLAL) and KLAL, of which the most prevalent etiology was SJS. After 43.4 months of mean follow-up, almost 80% of patients required a subsequent keratoplasty to improve visual acuity (21). These findings suggest that even though SJS is a condition with poor ocular prognosis, combined corneal and limbal transplant require an aggressive and diligent immunosuppression with a group of drugs that includes a T cell inhibitor, and that Tacrolimus is favored over Cyclosporine as a T cell inhibitor.

## Targeting Ocular Autoimmune Responses In Sjs Patients

Ocular autoimmune inflammatory disorders tend to present a chronic course. The correct management of these conditions involves the use of a steroid sparing agent that modulates B cell lymphocytes effectively with antimetabolites. In 2000, a consensus panel established that there is good evidence for the use of systemic immunosuppressive medication for various ocular inflammatory disorders, including mucous membrane pemphigoid (MMP) (22). This is an autoimmune disorder characterized by antibody deposition on the cell basement membrane that commonly attacks the mucocutaneous tissues. In particular, ocular cicatricial pemphigoid (OCP) is the variant that causes a cicatricial conjunctivitis that ranges from subepithelial fibrosis to ankyloblepharon with severe loss of visual acuity, similarly to SJS. OCP is another immunobullous disorder caused by an adaptive immune component that functions as an analog to explain the management of the autoimmune component of SJS patients, since there is scarce literature concerning the ocular management of the autoimmune responses of SJS patients. The treatment of MMP/OCP is through systemic immunosuppression, and the medications most frequently used are Dapsone, Mycophenolate, Methotrexate, Cyclophosphamide, and Rituximab (23). A proposed scheme is to start with tapered oral Steroids for 3 months, along with Mycophenolate 1-g BID. The use of mycophenolate is advised for its good efficacy at inhibiting not only T cells, but also B lymphocytes, in addition to presenting a safe toxicity profile and an easy dosing for the patient (24). For management of high-risk PK, a prospective, randomized trial compared the use of topical and systemic steroids with or without addition of 2 g of Mycophenolate/day for 6 months. At 1 year, the authors observed a statistical difference in the rate of immune reactions in 11% of those treated with MMF as opposed to 33% in the control group (*P* = 0.03) (25, 26). After 3 years of mean follow up no immune reactions were seen in 83% of MMF treated patients, in contrast to 64.5% in the control group (*P* = 0.04), and the rate of corneal rejection was much higher in the control group (78%), than the MMF group (20%) (25, 26). This data supports that MMF is a safe and effective drug to attenuate the autoimmune responses that OCP and SJS patients commonly present.

## Dual Immunomodulatory Approach For Sjs Patients Undergoing Surgery

In total, an immunologic approach for corneal or limbal stem cell transplants on SJS requires modulation of the alloimmune and autoimmune response in order to fully attenuate the inflammatory response to the corneal graft and avoid failure of therapy (Figure 1). As described, one component is the auto antigen surface disease, which is controlled with Mycophenolate and on the other hand one has to address the allotransplant immune response that attacks the living related and/or cadaveric KLAL. For this, a T cell inhibitor, preferably Tacrolimus should be used. The proposed immunosuppression regimen for SJS patients, as depicted by the Cincinnati group, should be as follow (21): At the time of surgery, solumedrol 500-1,000 mg, with immediate implementation of Tacrolimus 4 mg twice a day, and MMF 1 g twice a day. Prophylactic drugs such as valacyclovir and trimethoprim-sulfamethoxazole must be strongly considered. Tacrolimus levels must be measured at 3 months, and the optimal serum concentration is 8-10 ng/ml. Systemic prednisone is started with high initial doses at time of surgery, typically with intravenous Solumedrol 500-1,000 mg, followed by prednisone 1 mg/kg/day in the post-operative. Once inflammation is fully suppressed, at about one to 2 months, tapering of the steroid can begin. Importantly, steroid sparing therapy should be kept for a long period of time, similarly to a solid organ transplant.

## Considerations For Systemic Immunosuppression Medications

Of the presented groups of drugs, corticosteroids are a tempting resource to manage these patients, since these are highly effective, however they carry a myriad of adverse effects. For immunosuppressive outcomes on graft transplantation, the usual dose is 60 ± 20 mg of oral prednisone, which are highly effective; though the side effects are hyperglycemia, bone damage, weight gain, altered cognition, amid many others that should be screened at every clinic visit. In order to tackle the adverse effects, alternate day corticosteroids dosing is a possible solution, but corticosteroid-sparing agents should always be pondered. The T cell inhibitors function by inhibiting calcineurin activation, *via* binding to different proteins to block T cell proliferation. Cyclosporine A (CsA) works by binding to cyclophilin. It is generally dosed from 2 to 5 mg/kg. Known side effects are nephrotoxicity, high blood pressure, gingival hyperplasia and hirsutism. Tacrolimus binds to FK506-binding protein 12 (FKBP12) to form a complex that inhibits calcineurins. Thus, the initial phase of T cell activation is blocked, resulting in inhibition of T-lymphocyte signal transduction and IL-2 transcription (27). Tacrolimus has been reported to entail fewer systemic side effects than CsA, and these include nephrotoxicity in addition to high blood pressure and dyslipidemias as well but, unlike cyclosporine, these are more rare (28). Tacrolimus may cause diabetes and peripheral neuropathies, as well. It is generally dosed with 4-5 mg daily and monitoring should include creatinine; complete blood count (CBC); liver functions testings (LFT), blood pressure and cyclosporine/tacrolimus serum levels, which should range from 70 to 180 ug/L and 8-10 ng/mL, respectively, and ought to be strictly controlled. Finally, Mycophenolate mofetil inhibits the synthesis of guanosine nucleotides, resulting in selective inhibition of T- and B-lymphocyte proliferation. MMF is generally dosed with 2.0-3.0 g per day and its main side effects are diarrhea and bone marrow suppression. Therefore, monitoring is advised with CBC and LFT.

## Perioperative Management Of Sjs Patients

It is important to note that the management of the ocular surface of SJS patients is complex since lid anomalies, such as trichiasis, distichiasis, and/or lid entropion can be present and should be addressed prior to the corneal or limbal stem cell transplant. Two additional characteristics should be investigated: whether the bulbar conjunctiva is lubricated or significantly dry and if there is posterior eyelid margin keratinization. These difficulties can be resolved with mucous membrane grafting (MMG) if the eye is significantly dry and there is posterior eyelid margin keratinization. This procedure also helps improve severe dry eye, since the minor salivary glands that are present in the labial mucosa can be harvested and increase tear production. A recent multicentre study presented satisfactory long-term outcomes for 17 patients with SJS (29). Another series of SJS patients with lid related keratopathy observed that MMG and prosthetic replacement of the ocular surface ecosystem (PROSE) placement significantly increased long term vision, as compared to conservative treatment with medical management (30). Consequently, prior to performing the ocular surface reconstruction, a multidisciplinary approach should be considered with a nephrologist and an oculoplastic team. Post-operative management encompasses the use of topical steroids to reduce the ocular surface inflammation and prevent rejection from the graft, as well. Frequent tear substitution should be implemented with preservative free artificial tears or ideally serum tears or plasma rich in growth factors, since these have a myriad of growth factors and contribute to stabilization and nurturing of the ocular surface (31). Unfortunately, KLAL procedures in SJS have a high rate of failure due to different complications that may lead to a poor visual acuity, and these should be communicated with the patient.

## The Future Of Local Target Oriented Immune Regulation For Sjs Patients

**Figure 2:**
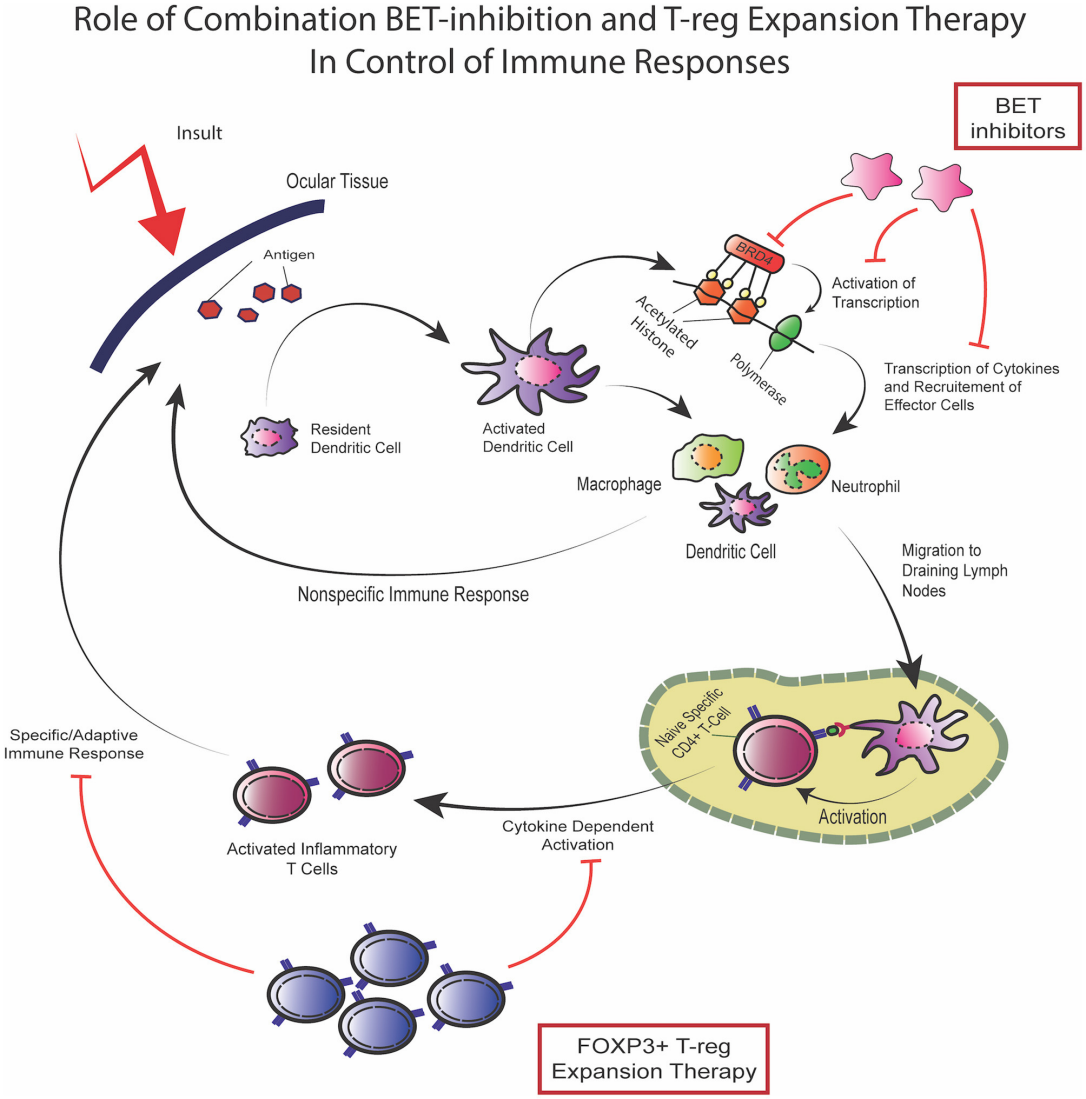
Combination of BET inhibition and T-reg expansion to target immune responses. In response to the presentation of novel antigens, as in the case of an ocular surface transplant, the resident immune cells activate and, through bromodomain-regulated activation of transcription, produce inflammatory cytokines which leads to recruitment of effector cells which carry out non-specific innate immune response. In addition, activated immune cells may also function as antigen presenting cells through MHC-II expression and migrate to draining lymph nodes where activation of antigen specific CD4+ T-cells takes place resulting in cytokine dependent specific immune response activation of the adaptive immune response. Dual therapy using BET-inhibitors mainly targeting the cytokine-dependent innate response along with T-reg expansion therapy targeting the activation of the adaptive response can potentially control immune responses and suppress inflammatory disease. The two modalities have been shown to not interfere with one another and can effectively provide a compound strategy for inflammatory control.

In 1995, Sakaguchi et al. discovered a population of CD4+ cells, which were termed regulatory T-cells (T-regs) (30, 31). CD4+FoxP3+ Tregs play integral roles in maintaining immune homeostasis, particularly through suppressing the immune response and modulating effector inflammatory cells (32–34). Importantly, identification of T-regs in organ transplants have implicated them as being important in graft tolerance (35). Downregulation of the inflammatory mechanisms is achieved *via* stimulation of inhibitory cytokines (34–36), reduction of effector inflammatory molecules (34, 37), and inhibition of dendritic cells (34, 38). Regulation of IL-2 reduces the levels of this cytokine to limit availability for conventional T-cell activation, since low doses of IL-2 selectively favor activation of T-regs over effector/conventional T cells (34, 39). Infusion of T-regs have shown promising results in different types of solid organ transplantation (40–42). Recently, our group developed a new approach to expand T-regs *in vivo* by targeting TNF receptor superfamily 25 (TNFRSF25) and CD25 using a TL1A-Ig fusion protein together with low dose IL-2 (43, 44). This strategy showed promise by demonstrating an impressive T-reg expansion in donor mice which ameliorated graft vs. host disease (GVHD) in pre-clinical models (45, 46). Epigenetic readers of histone acetylation can regulate transcription of genes involved in inflammation (43). Bromodomain and extra-terminal (BET) proteins which affect acetylation can be targeted using bromodomain and extra-terminal protein inhibitors (BETi). The BETi I-BET762 and JQ1 showed anti-inflammatory properties by disrupting the expression of pro-inflammatory cytokines in macrophages and suppressing genes involved in T cell-mediated pro-inflammatory functions (47). These have shown efficacy in a variety of inflammatory conditions (48–52). There have also been recent studies to investigate the use of bromodomain inhibitors to suppress responses against allo-antigens in transplantation (43). Such approaches provide the promise for developing novel platforms for new therapeutic options. Our group recently proposed a combinational strategy of BETi combined with T-reg expansion therapy and in murine models of allogeneic hematopoietic stem cell transplant, did not interfere with one another but together suppressed GVHD (43). In the future, this combinatorial platform could be considered for application to SJS patients to downregulate allo and auto immune responses following transplant (Figure 2).

## Conclusion

SJS is a rare, but highly morbid disease. Although new genetic associations of drugs are being recognized, the incidence of SJS has not dramatically changed, and its consequences are severe. On the eye, it commonly affects the ocular surface to the extent that it may need to undergo corneal surface reconstruction, with possible corneal or limbal stem cell transplantation to improve visual acuity. This presents an immunologic challenge, which should be managed with a dual approach to down regulate the immune response by using a T cell inhibitor and an antimetabolite (Tacrolimus and Mycophenolate are effective and safe choices) in addition to tapered systemic steroids. Further studies with diverse drugs, including monoclonal antibodies, are warranted to improve graft rejection outcomes. The future of immunosuppression for graft transplant involves local target oriented immune regulation *via* T-reg modulation and epigenetic mechanisms. These offer SJS patients and others promising opportunities to tackle the high risk of corneal rejection.

## Data Availability Statement

The original contributions generated for the study are included in the article/supplementary material, further inquiries can be directed to the corresponding author/s.

## Ethics Statement

Written informed consent was obtained from the individual(s) for the publication of any potentially identifiable images or data included in this article.

## Author Contributions

MS: collection, analysis and interpretation of data, manuscript preparation, and critical reading, edition of figures. HM: manuscript preparation and critical reading. RBL: interpretation of data and manuscript preparation. VLP: senior author of the review, involved in all aspects of this study. All authors contributed to the article and approved the submitted version.

## Conflict of Interest

RBL is a compensated consultant/advisory board member for and equity holder in Heat Biologics. VLP has worked as a compensated consultant for Alcon, Eyegate, Oculis, Novartis, Trefoil, Quidel, Dompe and is a board member of OBTears. The remaining authors declare that the research was conducted in the absence of any commercial or financial relationships that could be construed as a potential conflict of interest.
